# Induction of Nitric Oxide and Its Role in Facial Nerve Regeneration According to the Method of Facial Nerve Injury

**DOI:** 10.3390/antiox13060741

**Published:** 2024-06-19

**Authors:** Yeon Ju Oh, Dong Keon Yon, Yong Sung Choi, Jinseok Lee, Joon Hyung Yeo, Sung Soo Kim, Jae Min Lee, Seung Geun Yeo

**Affiliations:** 1 Department of Medicine, College of Medicine, Kyung Hee University Medical Center, Seoul 02447, Republic of Korea; 5duswn1203@khu.ac.kr; 2 Center for Digital Health, Medical Science Research Institute, Kyung Hee University School of Medicine, Kyung Hee University Medical Center, Seoul 02447, Republic of Korea; yondg@khu.ac.kr; 3 Department of Pediatrics, Kyung Hee University School of Medicine, Kyung Hee University Medical Center, Seoul 02447, Republic of Korea; feelhope@khu.ac.kr; 4 Department of Biomedical Engineering, Kyung Hee University, Seoul 02447, Republic of Korea; gonasago@khu.ac.kr; 5 Public Health Center, Danyang-gun 27010, Republic of Korea; joonhyungyeo@gmail.com; 6 Department of Biochemistry and Molecular Biology, College of Medicine, Kyung Hee University, Seoul 02447, Republic of Korea; sgskim@khu.ac.kr; 7 Department of Otorhinolaryngology Head & Neck Surgery, Kyung Hee University School of Medicine, Kyung Hee University Medical Center, Seoul 02447, Republic of Korea; sunjaesa@hanmail.net

**Keywords:** nerve, facial nerve, degeneration, regeneration, nitric oxide

## Abstract

Nitric oxide (NO) is an important molecule in cell communication that also plays an important role in many biological processes. Given the dual role of NO in nerve degeneration and regeneration after facial nerve injury, we sought to delve deeper into its role through a systematic literature review. A comprehensive review of the literature employing SCOPUS, PubMed, Cochrane Library, EMBASE, and Google Scholar databases was conducted to evaluate the induction and role of NO in neurodegeneration and regeneration after facial nerve injury. From the 20 papers ultimately reviewed, the central findings were that neuronal nitric oxide synthase(nNOS), endothelial nitric oxide synthase (eNOS), and induced nitric oxide synthase (iNOS) increased or decreased depending on the method of facial nerve damage, damaged area, harvested area, and animal age, and were correlated with degeneration and regeneration of the facial nerve. Research conducted on rats and mice demonstrated that NO, nNOS, eNOS, and iNOS play significant roles in nerve regeneration and degeneration. However, the relationship between nerve damage and NO could not be defined by a simple causal relationship. Instead, the involvement of NOS depends on the type of nerve cell, source of NO, timing, and location of expression, age of the target animal, and proximity of the damage location to the brainstem. Consequently, nNOS, eNOS, and iNOS expression levels and functions may vary significantly.

## 1. Introduction

### 1.1. Facial Nerve

The facial nerve is a mixed nerve that has four main functions: motor, secretion (parasympathetic), taste, and sensation. The motor nerve is responsible for movement of the muscles of the face and neck; the parasympathetic nerve component is responsible for secretion of the lacrimal gland and the salivary gland; special sensory nerves in the anterior two-thirds of the tongue senses taste; and the general sensory nerve is responsible for deep sensation in the auricle, posterior wall of the external auditory canal, ear lobe, and facial soft tissues [1]. The upper facial muscle is innervated bilaterally; thus, in the event of an upper lesion on one side of the motor nucleus, paralysis manifests mainly in the facial muscles around the mouth and lower face, whereas facial muscles around the forehead and eyes typically remain unaffected. Conversely, in the case of a unilateral motor nucleus lesion, paralysis occurs throughout the facial muscles of the upper and lower face on the affected side [2]. Among cranial nerves, the facial nerve is the most susceptible to damage owing to its long anatomical path within the skull and its superficial location outside the skull. It can be damaged by physical injury (e.g., traffic accidents), pressure caused by tumor growth, and surgical resection and infection, among other insults [3]. Severe damage or amputation of the facial nerve can damage the cornea, prevent eye closure, and cause difficulty in the early stages of swallowing food, making it difficult to retain food in the mouth. It can also leave patients with serious deformations in facial appearance. Thus, facial nerve paralysis has a direct impact on quality of life, with patients experiencing this condition exhibiting a higher incidence of depression and facing substantial disruptions in social activities [4]. Until recently, microsurgery was the preferred clinical solution following facial nerve damage, but the reality is that it is difficult to achieve complete recovery of motor function through surgery [5].

### 1.2. Free Radicals and NO

A free radical is a molecule that is unstable owing to an imbalance in its electron configuration. This instability arises when oxygen, absorbed from the air, loses an electron during physiological processes in the body, becoming a reactive species unable to pair its unpaired electron. There are many types of free radicals, with the best-known being reactive oxygen species (ROS) and reactive nitrogen species (RNS) [6] (Table 1). Free radicals of ROS include superoxide (O_2_^−^), hydroxyl (^−^OH), peroxyl (LOO*), and alkoxyl (LO*) radicals [6] (Table 1).

**Figure 1:**
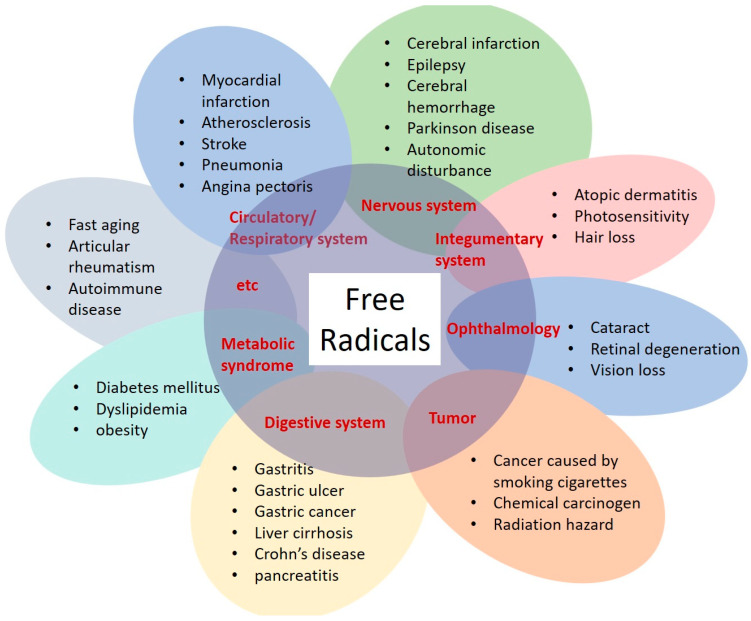
Diseases related to free radicals.

Free radicals, including ROS and RNS, have been implicated in a wide range of health issues. In addition to being associated with aging and cancer, these free radicals contribute to aspects of skin aging, including wrinkles and age spots; eye diseases such as cataracts; brain diseases such as Parkinson’s and Alzheimer’s diseases; and cardiovascular conditions such as arteriosclerosis and heart disease [7,8]. Free radicals also contribute to various other disorders, including allergic and autoimmune diseases such as atopic dermatitis and rheumatoid arthritis; as well as diabetes, hair loss, hearing loss, obesity, gastritis and liver lesions [7,8] (Figure 1).

RNS are generally regarded as a subgroup of ROS. One prominent RNS is nitric oxide (NO), which was initially identified as an air pollutant emitted by automobiles and factories and subsequently discovered to be synthesized in mammals in 1987. NO is a colorless, odorless gas composed of one nitrogen (N) and one oxygen (O) [9,10]. NO is synthesized from L-arginine by the enzyme NO synthase (NOS). There are three types of NOS: eNOS (endothelial NOS), nNOS (neuronal NOS), and iNOS (inducible NOS), each with distinct expression, regulation, and tissue distribution [11,12]. nNOS and eNOS, generally continuously expressed, are sometimes termed constitutive NOS (cNOS), whereas iNOS is transcribed and expressed (i.e., induced) only in response to specific stimuli. NO production by cNOS isoforms is closely regulated by intracellular calcium concentration (calcium/calmodulin-dependent), which determines the amount of NO produced. The result is the production of minute quantities of NO (picomole-nanomole) briefly when necessary, facilitating various physiological functions mediated by the cGMP pathway [13,14]. Unlike eNOS and nNOS, which produce NO for only a short period of time after activation by calcium and calmodulin [15,16], iNOS produces NO independent of calcium and calmodulin, generating significant NO amounts (micromole level) more slowly than cNOS and over extended periods (hours to days) [17,18].

Though also expressed in skeletal muscle and lung epithelial cells, nNOS is mainly expressed in nervous tissue, where plays a crucial role in neurotransmission; it can also contribute to nerve damage in pathological conditions by regulating synaptic plasticity and signaling of nerve cells [19,20]. eNOS, expressed primarily in vascular endothelial cells but also in cardiac muscle cells and pyramidal cells of the hippocampus [21], is involved in vasodilation, neovascularization, inhibition of platelet aggregation, and erectile function through relaxation of the corpora cavernosa [22]. iNOS produces NO in some cells of the nervous system (e.g., astrocytes and microglia), but is also expressed in dendritic cells, macrophages, vascular smooth muscle, endothelial cells, hepatocytes, osteoclasts, and epithelial cells.

iNOS expression is regulated at the transcriptional level in response to stimuli such as cytokines [23]. NO produced by iNOS has both physiological functions and pathological roles [24]. Known inducers of iNOS include interferon (IFN)-γ, tumor necrosis factor (TNF)-α, interleukin (IL)-1, the bacterial endotoxin lipopolysaccharide (LPS), and CpG motifs [25], which primarily play roles in immune response regulation and cytotoxicity [26]. iNOS transcription is known to be regulated by NF-kB, which itself is subject to negative feedback regulation by NO via a mechanism involving post-translational modification of the NF-kB suppressor, IkB, which prevents excessive NO production [27].

Approaches for inhibiting NO synthesis are diverse and include treatment with (1) arginine analogues; (2) Nicotinamide Adenine Dinucleotide Phosphate (NADPH) inhibitors such as diphenyleneiodonium; (3) calmodulin antagonists such as trifluoperazine, chlorpromazine, calmidazolium, W7, and W13; (4) inhibitors of BH4 synthesis such as diamino-6-hydroxypyrimidine (DAHP), methotrexate, and N-acetyl-5-hydroxytryptamine; (5) NO for feedback inhibition; and (6) substances that react with heme such as carbon monoxide and methylene blue [28,29] (Table 2).

## 2. Nervous System and NO

NO is responsible for various functions in the central nervous system (CNS). Within the brain, nNOS (type I) is found in neurons, iNOS (type II) is found in glial cells, and eNOS (type III) is primarily found in endothelial cells. The main actions of nNOS in the CNS can be divided in four categories: neurotoxicity, neuroprotection, synaptic plasticity, and modulatory activity [28].

Neurotoxicity, which occurs under abnormal pathological conditions such as hypoxia, is caused by excessive secretion of glutamate, which binds to NMDA receptors, promoting Ca^2+^ influx into neurons and consequent activation of NOS and synthesis of NO [28,30]. NO reacts with superoxide (O_2_^−^) to form peroxynitrite (ONOO-), which causes cytotoxicity. NO also inhibits glycolysis by nitrosylating several enzymes, including PKC (protein kinase C) and GADPH (glyceraldehyde-3-phosphate dehydrogenase); causes DNA denaturation and strand breakage; and activates poly (ADP-ribose) polymerase (PARS), leading to massive energy depletion and cell death. NO also reacts with iron in heme or non-heme (Fe-S) complexes, causing other deleterious effects, such as inhibition of glycolysis. The converse effect of NO—neuroprotection—is achieved by NO acting on NMDA receptors to inhibit glutamate binding and Ca^2+^ influx into cells [31]. Synaptic plasticity includes long-term potentiation (LTP) and long-term depression (LTD). LTP amplifies the effect of synaptic transmission, causing an increase in synaptic strength; it occurs mainly at the presynaptic nerve, postsynaptic receptor, and postsynaptic dendritic spine. LTD plays a crucial role in the learning and fine-tuning of motor movements within the cerebellum. Regulatory actions of NO include pain perception, memory formation, and learning processes. Notably, experimental inhibition of NOS results in an antinociceptive effect [28,32]. In the peripheral nervous system, NO generally functions as a neurotransmitter, mainly as an inhibitory neuromuscular neurotransmitter. Rather than acting alone, it relaxes muscles mainly by interacting with VIP (vasoactive intestinal polypeptide) or through co-transmission with ATP. In this capacity, it causes reflex receptive relaxation of the stomach, descending and tonic inhibition of the intestines, relaxation of the lower urinary tract (i.e., bladder neck and urethra), and relaxation of the male (cavernosal tissue and retractor penis muscle) and female (uterus) reproductive tract [28]. In the cardiovascular system, NO causes neurogenic vasodilation and regulates the vasoconstriction influence of sympathetic transmission; it also exerts a muscle-relaxing effect in the respiratory system. NO is also known to be involved in neurogenic vasodilation and pain perception in primary sensory neurons and is thought to play a role in motor neurons as well, although this is not firmly established [28,33].

Although NO has been studied in various disease contexts, the extent to which NO induces or contributes to neurodegeneration and regeneration after nerve injury is not yet well established, and there is a particular lack of information about the facial nerve, despite its importance.

**Figure 2:**
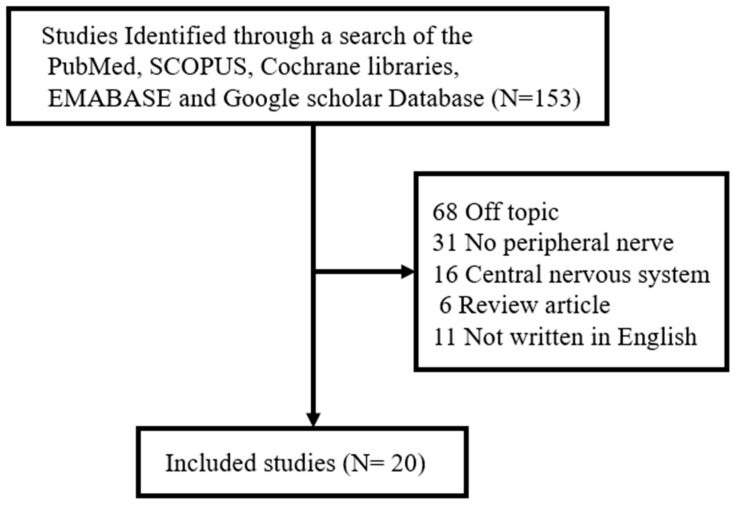
Review flow diagram.

Against this backdrop, we performed a literature review of studies examining recovery from facial nerve injury and effects of NO. To this end, one of the authors (J.H.Y) retrieved papers published between January 1994 and March 2024 from five electronic databases—PubMed, SCOPUS, Cochrane libraries, EMBASE, and Google scholar—based on the search terms, ‘facial nerve’, ‘nerve injury’, and ‘nitric oxide’, focusing on studies published in English, including prospective or retrospective studies on NO in the facial nerve, and studies on nerve degeneration and regeneration (in humans or animals). Exclusion criteria included (1) unpublished data, (2) review articles, (3) gray literature, (4) case reports, and (5) duplicate cases. A total of 20 studies out of 153 satisfied these exclusion/inclusion criteria (Figure 2).

## 3. Production of NO and Expression of NO-Related Factors after Facial Nerve Injury

### 3.1. Association of NO with Neurodegeneration (Table 3)

Studies have linked NO to neurodegeneration. For example, NO production was reported to be higher on the side with than without nerve damage. Immunohistochemical staining for NOS following transection injury to the facial nerve showed excess production of NO in axotomized motor neurons, with this increase in NO expected to play a causal role in neuronal cell loss [34]. Crush injury and administration of toxin (ricin) to the facial nerve have been found to enhance NADPH-d activity, with NO production by reactive astrocytes observed in the untreated group after injury [35]. These authors showed that increased expression of inflammatory cytokines IL-1β, TNF-α, IL-6, and lipopolysaccharide produced after induction of iNOS by facial nerve transection, and the overproduction of NO itself produced a neuro-destructive effect. Subjecting the facial nerve to crush injury and transection injury has been found to induce NADPH-d activity 7 days later, with this activity being higher at 14 days, but decreasing by 1 month [36]. The increased NADPH-d activity at 14 days was accompanied by a 70% reduction in motoneurons. NADPH-d staining after transection injury to the facial nerve showed the presence of NADPH-d positive motoneurons in the facial nucleus beginning 7 days after axotomy [37].

**Table 3:** Literature on the involvement of NO production in neurodegeneration after facial nerve injury.

Author	Study Design	Species and/or Sample	Nerve/Injury Methods	Detection Method	Target Gene(s)	Results/Conclusions
Yu, 1994 [34]	Animal study	Sprague-Dawley rats/FMN	Sciatic nerve, hypoglossal nerve, vagus nerve, facial nerve/ transection	NADPH-d histochemistry, NOS immunohistochemistry, cell count	NOS	After transection of peripheral nerves, NOS expression was significantly up-regulated in axotomized sensory ganglion cells, but not in corresponding motor neurons unless axon regeneration was prevented and ensuing neuron death became massive. /NOS expressed in axotomized motor neurons may play a causal role in neuronal cell loss through excess production of NO.
McElhaney et al., 1994 [35]	Animal study	Sprague-Dawley rats	Facial nerve/crush injury & toxin (ricin)	NADPH-d histochemistry	NOS	NADPH-d activity is increased in reactive astrocytes in response to ricin-induced degeneration of FMNs but not following untreated axotomy (saline injection). /Irreversible neuron injury resulting in neurodegeneration causes increased NO production by reactive astrocytes.
Kassa et al., 2007 [36]	Animal study	Wistar rats	Facial nerve/crush and transection injury	Western blotting (Bcl-2, P2X1, P2X2), immunohistochemistry (GFAP, OX-42, NADPH-d)	NOS	We found NOS induction at 7 days, with increment at 14 days and downregulation at one month. /We show in damaged motoneurons a parallel degree of induction of NOS and P2X1, which was weaker for both molecules following crush injury than after nerve resection. These findings indicate that P2X1 and NOS induction share not only temporal features, but also quantitative ones.
Che et al., 2000 [37]	Animal study	Sprague-Dawley rats	Facial nerve/transection	hybridization for PIN, NADPH-d staining	NOS	NADPH-d-positive motoneurons were found in the facial nucleus beginning 7 days after axotomy. /PIN may interact with NOS from 7 days post-operation.
Zhang et al., 2010 [38]	Animal study	Wistar rats	Facial nerve/transection	iNOS immunohistochemistry, cresyl violet staining, cell counting	NOS, nuclear factor (NF)-kB pathway	After facial nerve damage, cytokines, including IL-1β, TNF-α and IL-6, which have been shown to induce iNOS expression via different pathways, are upregulated in the facial nucleus. Erythropoietin treatment was associated with a significantly lower percentage of iNOS+ neurons compared with saline treatment at each time point, especially at 3 weeks after axotomy. /A high dose of erythropoietin attenuates the increase in iNOS expression in the facial nucleus after facial nerve transection, and thus may enhance the survival of FMNs.
Chen et al., 2008 [39]	Animal study	Guinea pig	Facial nerve/transection	nNOS immunohistochemistry, cresyl violet staining, cell counting	NOS	Facial nerve transection induced a significant increase in NO formation in the brainstem by 1 week in both MPSS- and saline-treated groups and lasted to the end of the study at 4 weeks. But MPSS treatment led to significantly lower numbers of NOS(+) neurons and NO levels in the brainstem throughout the initial 2 weeks post-operative survival period. /MPSS could delay the increase in NO formation after facial nerve transection and may thereby enhance the survival of motor neurons.
Ito et al., 2007 [40]	Animal study	Wistar (adult male)/FMN	Facial nerve/transection	NADPH-d histochemistry, cresyl violet histochemical staining of FMN	NOS	The number of surviving motoneurons in the ipsilateral FMN was significantly greater among TJ-23-treated rats than nontreated controls on day 56 following axotomy. /Orally administered TJ-23 is effective in reducing neuronal NADPH-d expression in FMN neurons after peripheral nerve axotomy.
Sakamoto et al., 2003 [41]	Animal study	Fischer 344 male rats	Facial nerve/avulsion injury	Western blot analysis, NADPH-d histochemistry, immunostaining	NOS	Treatment with adenovirus encoding GDNF, BDNF, or TGF-β2 after avulsion significantly attenuated the loss of lesioned facial motoneurons, and prevented the induction of NOS activity in these neurons. /The NOS inhibitors, nitroarginine and L-NAME prevent the induction of NOS activity and subsequent motoneuron death after avulsion.
Wang et al., 2009 [42]	Animal study	Sprague Dawley rats	Facial nerve/transection	Electrophysiological recordings, immunohistochemistry, ssDNA biomarker (DNA fragmentation), facial nucleus image analysis	NOS	Facial nerve repair+NOS inhibition promoted earlier and better axonal regeneration than facial nerve repair alone, /Peripheral nerve suture and/or treatment with NOS inhibitors helps maintain the homeostasis of oxidative stress-related biomarkers, especially nNOS, in neuronal cell bodies.
Yeh et al., 2017 [43]	Animal study	Sprague Dawley rats/FMN	Facial nerve/transection	GFAP immunofluorescence, OX-42 immunolabeling, NeuN immunolabeling	NOS, GFAP, OX-42	Inhibition of NOS with L-NAME had no effect on astrocytic and microglial reactions in the descending central facial tract. Inhibition of NO production significantly reduced microglial but not astrocytic reaction in the facial nucleus after neurorrhaphy. /NO is involved in the activation of microglia in the facial nucleus after facial neurorrhaphy.
Casanovas et al., 1996 [44]	Animal study	Sprague-Dawley	Facial nerve/transection	Nissl staining, cell counting, histochemical detection of DNA fragmentation, immunocytochemistry (cNOS, iNOS)	NMDA receptor, NOS	The NOS inhibitor L-NAME was also able to protect motoneurons from death, but to a lesser extent. /The potential overactivation of glutamate receptor in motoneuron death by axotomy should be considered, since this leads to increased release of NO which, in turn, may participate in neuronal damage. NO derived from activated astrocytes may have a role in promoting excitotoxic mechanisms in axotomized motoneurons.
Jacob et al., 1999 [45]	Animal study	Fischer 344 rats and FMN cell bodies of axons	Facial nerve/transection	Immunohistochemistry using an antibody to iNOS on tissue sections and slot blots	NOS	iNOS expression was increased ~12-fold in isolated blood vessels from old rats compared to vessels from adult animals. /Aging and injury differentially affect the expression of iNOS, and up-regulation of iNOS may be important for the availability of NO in the aged or injured nervous system.
Liu et al., 2006 [46]	Animal study	Wistar rats	Facial nerve/transection, (proximal; brain-stem surface, distal; stylomastoid foramen)	Light microscopy, TUNEL assay, electron microscopy, cell counting, immunoreactivity	NOS	Proximal axotomy upregulated NOS in the absence of a transient downregulation in the expression of calcineurin and Mn-SOD at 4 weeks after facial nerve injury. /Axotomy of the FMN revealed significant differences between central neurons projecting outside the CNS and neurons projecting within the CNS in their response to proximal and distal axonal injury, based on a model using rat RS neurons.
Che et al., 2000 [47]	Animal study	Sprague-Dawley rats	Facial nerve/transection	In situ hybridization, NADPH-d staining, retrograde tracing of axotomized neurons using WGA	PSD-95. CAPON, NOS	PIN mRNA was initially expressed and transiently increased frome 3 to 5 days and returned to the basal level at 7 days after axotomy in the motoneurons of the facial nucleus. NADPH-d positive motoneurons were found from 7 days post-operation in the facial nucleus. /Increased expression of PIN might inhibit mainly nNOS activity as well as all NOS activities and downregulate NO production. Upregulation of PIN mRNA may serve to enhance retrograde transport of factors needed for nerve regeneration.

Abbreviations: NO, nitric oxide; NOS, nitric oxide synthase; cNOS; constitutive nitric oxide synthase; iNOS, inducible nitric oxide synthase; eNOS endothelial nitric oxide synthase; nNOS, neuronal nitric oxide synthase; RNA, ribonucleic acid; mRNA, messenger RNA; DNA, deoxyribonucleic acid, ssDNA, single-stranded DNA; FMN, facial motor nucleus; NADPH-d, dihydronicotinamide adenine dinucleotide phosphate diaphorase; l-NAME, Nω-nitro-L-arginine methyl ester; L-NIL, L-N-(1-iminoethyl)-lysine hydrochloride; SMLT, S-methyl-L-thiocitrulline; 7-NI, 7-nitronidazole; NF-κB, nuclear factor kappa-light-chain-enhancer of activated B cells; IL-1β, interleukin-1beta; TNF-α, tumor necrosis factor-α; IL-6, interleukin-6; Bcl-2 B-cell leukemia/lymphoma 2 protein; ALS, amyotrophic lateral sclerosis; GFAP, glial fibrillary acidic protein; OX-42, anti-integrin alpha M [CD11b] antibody; NeuN, neuronal nuclear antigen; IANX, inferior alveolar nerve transection; TG, trigeminal ganglion; MDA, malondialdehyde; DXM, dexamethasone; TJ-23, Tokishakuyakusan; TUNEL, terminal deoxynucleotidyl transferase dUTP nick-end labeling; SOD, superoxide dismutase; CNS, central nervous system; ABC, avidin-biotin complex; H&E, hematoxylin and eosin; GAP-43, growth associated protein-43; PIN, protein inhibitor of neuronal nitric oxide synthase; GDNF, glial cell line-derived neurotrophic factor; BDNF, brain-derived neurotrophic factor, TGF-β2, transforming growth factor-beta 2; MK-801, dizocilpine maleate; NMDA, N-methyl-D-aspartate; MPSS, methylprednisolone sodium succinate.

**Figure 3:**
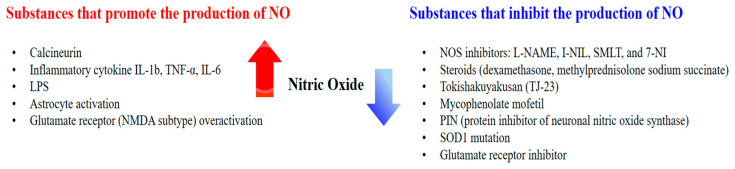
Substances that increase or inhibit the production of NO.

Several studies evaluating recovery from neuronal injury have been based on the correspondence between the degree of neuronal injury and the NO production index. The relationship between reduced NO production and increased motor neuron survival in response to drug treatment suggests that NO affects neuron damage. For example, immunohistochemical staining for iNOS following transection injury to the facial nerve showed that iNOS expression in the facial nucleus was higher on the injured than on the uninjured side [38]. Additionally, high doses of erythropoietin increased the survival of facial motor neurons and attenuated iNOS expression, whereas overproduction of NO had a neuro-destructive effect [38]. Similarly, immunohistochemical staining for nNOS following transection injury to the facial nerve showed that NO production in the brainstem was significantly higher on the injured than on the uninjured side [39]. In addition, MPSS was found to enhance the survival of facial motor neurons and delay NO formation. Transection injury to the facial nerve reduced NADPH-d expression in the TJ-23 (Tokishakuyakusan)-treated group, with the number of surviving motoneurons being significantly higher than in nontreated controls on day 56 [40]. Treatment with adenovirus encoding GDNF, BDNF, and TGF-β2 significantly attenuated the loss of lesioned facial motoneurons and prevented the induction of NOS activity in these neurons [41] (Figure 3).

The use of an NOS inhibitor to inhibit NO production has been found to increase the recovery rate of nerves following injury, further suggesting that NO is associated with neurodegeneration. These findings were supported by immunohistochemical staining for NOS following transection injury to the facial nerve. For example, axonal regeneration was increased following facial nerve repair and/or administration of an NOS inhibitor, suggesting that suturing of the peripheral nerve and/or treatment with NOS inhibitors helps maintain the homeostasis of oxidative stress-related biomarkers, especially nNOS, in neuronal cell bodies [42]. Similarly, L-NAME inhibition of NO production significantly reduced microglial but not astrocytic NOS in the facial nucleus after neurorrhaphy [43]. Administration of the NOS inhibitors nitroarginine and L-NAME following avulsion injury to the facial nerve was found to reduce motoneuron death [41].

Immunocytochemical analysis of cNOS and iNOS after a transection injury to the facial nerve showed that reactive astrocytes around axotomized motoneurons in young animals were positive for iNOS, with the NOS inhibitor L-NAME able to protect motoneurons from death [44]. The potential overactivation of the glutamate receptor was associated with an increase in NO release, which may, in turn, participate in neuronal damage. NO derived from activated astrocytes may promote excitotoxic mechanisms in axotomized motoneurons. Immunohistochemical analysis of iNOS after transection injury to the facial nerve showed iNOS expression was ~12-fold higher in isolated blood vessels from old than from adult rats. These findings suggested that both aging and injury can affect the expression of iNOS, with its up-regulation being important for the availability of NO in aged or injured nervous systems [45]. When comparing the results of the proximally axotomy (brain stem surface) group and the distally axotomy (stylomastoid foramen) group, nNOS increased more and more loss of facial motoneurons occurred at 1 week after injury in the proximally axotomy group [46].

NADPH-d activity was observed after transection injury to the facial nerve, with NADPH-d positive motoneurons in the facial nucleus observed beginning 7 days after surgery. Upregulation of mRNA encoding protein inhibitor of neuronal nitric oxide synthase (PIN) is regarded as necessary for nerve regeneration, as increased expression of PIN might downregulate NO production and inhibit all NOS activities, especially nNOS [47].

### 3.2. NO Is Involved in Nerve Regeneration (Table 4)

Thermal damage to the facial nerve was found to reduce nNOS expression, as determined immunohistochemically [48]. Transection injury to the facial nerve reduced the expression of nNOS in permanently denervated muscle fibers for up to 24 weeks but did not alter the expression of eNOS or iNOS [49]. Both of these studies showed that nNOS expression was reduced in areas where nerves had not regenerated, suggesting that nNOS is related to nerve regeneration.

**Table 4:** Literature on the involvement of NO production in nerve regeneration after facial nerve injury.

Author	Study Design	Species and/or Sample	Nerve/Injury Methods	Detection Method	Target Gene(s)	Results/Conclusion
Aslan A. et al., 2005 [48]	Animal study	Guinea pigs	Facial nerve/thermogenesis (drilling)	H&E staining, Solochrome cyanine staining, nNOS immunostaining	NOS, P2X receptor, Ca^2+^ signaling, NO-cGMP-PKG pathway	Axonal fibers in thermally damaged facial nerves exhibited scattered, localized edema, as well as a decrease in both the intensity of nNOS and the number of nNOS+ cells. /Thermal damage of the facial canal may cause deterioration in nerve conduction to some extent, as evidenced by changes in nNOS activity and the thickness of myelin fibers.
Tews DS, et al., 1997 [49]	Animal study	Wistar rats	Facial nerve/transection	immunohistochemistry (nNOS, iNOS, eNOS)	NOS	nNOS expression was downregulated in permanently denervated muscle fibers, a loss that persisted to week 24, without a change in eNOS or iNOS. nNOS loss was similarly observed in denervated and immediately reinnervated muscles, but nNOS levels returned to normal at week 10. /Downregulation of nNOS and subsequent decreases in NO production may contribute to neurodegeneration through apoptosis.
Wong PT et al., 1995 [50]	Animal study	Wistar rats/ipsilateral FMN	Facial nerve/compression	Eyeblink reflex, vibrissae movement, nasal tip orientation, NOS radiometric assay (using arginine), NADPH-d histochemistry	NOS	Following facial nerve compression injury, complete paralysis was confirmed after 5 days, and recovery of function was confirmed in behavioral experiments between days 20 and 40. Endothelial NOS activity increased at day 7 after injury in the ipsilateral FMN. nNOS increased between days 21 and 42, peaking on day 35 and subsequently decreasing compared with sham-operated animals. The period of increased eNOS coincided with the period of complete paralysis, and the period of increased nNOS coincided with the period of recovery of nerve function. /eNOS may be involved in neurodegeneration, whereas nNOS may be involved in nerve regeneration.
Rossiter JP, et al., 1996 [51]	Animal study	Sprague–Dawley rats	Facial nerve/transection	Nissl-staining, ISEL-labeling, NADPH-d histochemistry, fluorescence photomicrographs of facial GFAP immunocytochemistry	NOS	NO increased after DNA fragmentation and neuronal cell death had already shown significant progression. /Increased NADPH-d activity is not an initial causal factor in the death of facial motor neurons.
Mariotti R, et al., 1997 [52]	Animal study	Wistar male rats	Facial nerve/transection	NADPH-d histochemistry	NOS	Marked apoptotic phenomena in facial motoneurons at a time when no NADPH-d histochemical postivity was evident suggests that NO may not represent an ubiquitous necessary event preceding motoneuronal cell death after injury. /Death of immature motoneurons disconnected from their target and NOS induction may represent unrelated phenomena.
Mariotti R et al., 2002 [53]	Animal study	Mice carrying a mutated SOD1 gene and wild-type mice/FMN	Facial nerve cutting injury (left buccal and mandibular branches)	Nissl staining, NADPH-d histochemistry	NOS, SOD1	Facial nerve injury caused a decrease in motor neurons in both wild-type and transgenic (SOD1 mutant) mice on the lesion side, a decrease that was more evident in transgenic than in wild-type mice. NADPH-d positivity was evident in wild-type mice 2–3 weeks after injury, but was barely detectable in transgenic mice. /The NOS induction system affects neuronal self-defense mechanisms.

Abbreviations: NO, nitric oxide; NOS, nitric oxide synthase; NADPH-d, dihydronicotinamide adenine dinucleotide phosphate diaphorase; cNOS; constitutive nitric oxide synthase; iNOS, inducible nitric oxide synthase; nNOS, neuronal nitric oxide synthase; eNOS, endothelial nitric oxide synthase; WGA, wheat germ agglutinin; PSD-95, post-synaptic density-95; CAPON, carboxyl-terminal PDZ ligand of nNOS; RNA, ribonucleic acid; mRNA, messenger RNA; DNA, deoxyribonucleic acid; ssDNA, single-stranded DNA; cGMP, cyclic guanosine monophosphate; PKG, protein kinase G; FN, facial nucleus; FMN, facial motor nucleus; GFAP, glial fibrillary acidic protein; ISEL, in situ end labeling; SOD, superoxide dismutase; H&E, hematoxylin and eosin; PN, post-natal day.

**Figure 4:**
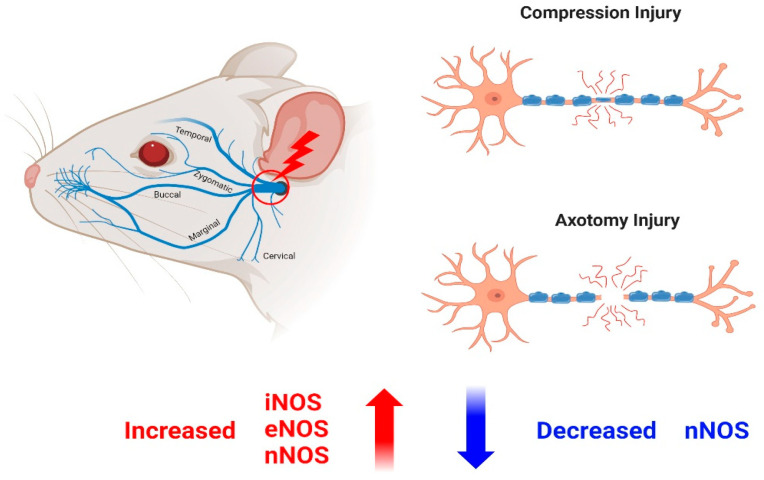
Production of NO in the context of facial nerve degeneration and regeneration after facial nerve injury. When the facial nerve was injured, the expression levels of nNOS, eNOS, and iNOS increased. However, in some experiments, nNOS expression showed a decrease depending on the experimental conditions.

The effects of compression injury on the facial nerve on NOS and NADPH-d were also assessed. The period of complete paralysis coincided with the period of increased eNOS expression, whereas the period of recovery of nerve function coincided with the period of increased nNOS expression. These findings suggested that eNOS may be involved in neurodegeneration, whereas nNOS may be involved in nerve regeneration [50] (Figure 4).

Although NADPH-d activity following transection injury to the facial nerve was found to be increased on the damaged side, neuronal cell death had already progressed to a significant extent, suggesting that increased NADPH-d activity is not a causal factor in the death of facial motor neurons [51]. Similarly, the period of marked apoptosis after transection injury to the facial nerve coincided with an absence of NADPH-d positivity [52]. These results suggested that neurodegeneration was not associated with increased NADPH-d activity after nerve injury, as the latter did not coincide with the period of neuronal call death [51,52]. Therefore, NO was not an initial cause of nerve damage. A comparison of wild-type and transgenic (SOD1 mutant) animals after cutting injury to the facial nerve showed that motor neuron loss was greater and NADPH-d positivity lower in the transgenic than in the wild-type group. Thus, NO is likely unrelated to neuronal cell death; rather, the NOS induction system is related to the neuronal self-defense mechanism [53].

### 3.3. Factors That May Affect NO Production after Nerve Injury

(1) Age of experimental animals

Experimental results may depend on the age of the experimental animals. For example, immunocytochemistry following transfection injury to the facial nerve showed that reactive astrocytes around axotomized motoneurons were positive for iNOS in neonatal (3-day-old) rats, but negative in adult (2-month-old) rats [44]. Furthermore, the interval from transection injury to NADPH-d positivity was 4 days in 1- and 2-week-old rats, but 7 days in 5-, 8-, and 12-week-old rats, indicating that the time interval from injury to NO expression was shorter in younger than in older animals [52]. In addition, immunohistochemical analysis showed that iNOS expression was ~12-fold higher in isolated blood vessels from older than from adult rats [45].

(2) Types of NOS (nNOS, eNOS)

The roles played by NOS in neurodegeneration and recovery may also depend on the type of NOS. Specifically, eNOS may be involved in neurodegeneration, whereas nNOS may be involved in nerve regeneration [50].

(3) Target cells (e.g., astrocytes, microglia, neuronal cells, oligodendrocytes)

Measurements of NO may also depend on cell type. Immunocytochemistry showed that iNOS was expressed by reactive astrocytes surrounding axotomized motoneurons [44], whereas other studies have linked NO expressed in microglia rather than astrocytes to nerve damage. After transection injury to the facial nerve, the levels of expression of GFAP, an indicator of astrocytic activation, and of OX-42, an indicator of microglial activation, were found to be higher in the facial nucleus of the injured group. Additionally, administration of an NOS inhibitor after injury reduced microglial activation, while increasing astrocytic reaction, suggesting that NO may be involved in microglial activation after nerve injury [43]. Intraneural injection of the toxin ricin, which causes acute neurodegeneration, resulted in NADPH-d staining not only in neurons but also in reactive glial cells. Based on the results of these studies, the authors argued that it cannot be concluded that simply increasing NO in neurons after nerve damage causes neurodegeneration, and that the NO that causes neurodegeneration is produced by reactive glial cells, not neurons [35].

(4) Location of injury

The distance between the brainstem and the site of damage can also affect NO production. nNOS immunoreactivity was compared in rats that underwent proximal axotomy on the brainstem surface or distal axotomy from the stylomastoid foramen. NO production was higher, and more facial motoneurons lost in rats that underwent proximal than distal axotomy, suggesting that NO was more cytotoxic when a proximal axotomy was performed [46].

## 4. Conclusions

Various studies have evaluated the relationship of NO expression with nerve regeneration and degeneration after facial nerve injury. To summarize, damage to the facial nerve through crushing, cutting, or removing a piece of the nerve was associated with increases in the levels of nNOS, eNOS, and iNOS expression. Under several experimental conditions, however, nNOS decreased after these types of facial nerve damage. In addition, NO expression after facial nerve injury was associated with nerve regeneration or neurodegeneration, depending on the experimental conditions and methods.

Injury to the facial nerve was associated with increased NOS activity in the ipsilateral facial nucleus along with neuronal cell loss, although these results differed among studies. Depending on the method and/or degree of damage, however, a time interval was required for NOS to appear after facial nerve damage; thus, the appearance of NOS depended on the time it was measured after facial nerve damage. Moreover, differences were observed in the types of cells that expressed NOS (e.g., endothelial cells, neuronal cells, microglia, and astrocytes) and the location of NOS expression, with the cells and nerve regions selected differing among studies. Results may also depend on the type of NOS expressed (e.g., iNOS, nNOS, and eNOS); when NO expression was confirmed by NADPH-d positivity, it was not possible to identify the type of NOS involved. It was also unclear whether both NADPH-d and NOS accurately reflect the NO produced within cells. Due to these limitations, it was not possible to accurately determine the effect of NO on facial nerve damage.

The current experimental method of determining increases in NO activity after facial nerve damage was unable to assess whether the increased NO activity was related to nerve damage or the process of recovering the damaged nerve. Thus, despite studies showing similar results, their conclusions differed. Clarification of these differences requires more detailed examination and further research.

It has been found that free radicals derived from the activity of eNOS, iNOS, and nNOS are involved in nerve regeneration and degeneration after facial nerve injury. Whereas research on the role of nNOS in nerve regeneration and degeneration has yielded mixed results, research on eNOS points to an exclusive role in nerve degeneration. In any case, the link between nerve damage and NO is not a simple cause-and-effect relationship because the expression of iNOS, eNOS, and nNOS varies depending on the type of nerve cell used in the study, the timing and duration of NOS expression, the expression site, the age of the experimental animal, the injury method, and the location of the injury.

## Figures and Tables

**Table 1:** List of ROS and RNS [6].

	Free Radical	Symbols	Non-Radical	Symbols
Reactive oxygen species	Superoxide radicals	O_2_^−^	Hydrogen peroxide	H_2_O_2_
	Hydroxyl radicals	^−^OH	Singlet oxygen	^1^O_2_,
	Peroxyl radical	LOO▪	Lipid hydroperoxide	LOOH
	Alkoxyl radical	LO▪	Peroxynitrite	ONOO^−^
			Hypochlorous acid	HOCl
			Ozone	O_3_
Reactive nitrogen species	Nitric oxide	NO	S-nitrosothiols	
	Nitrogen dioxide	NO_2_	Peroxynitrite	ONOO^−^
			Nitroxyl anion	NO^−^
			Nitrate	NO_3_^−^
			Nitrosonium cation	NO^+^
			Dinitrogen trioxide	N_2_O_3_
			Dinitrogen tetroxide	N_2_O_4_
			Nitryl chloride	NO_2_Cl
			Nitrous acid	HNO_2_

**Table 2:** Summary of the NO inhibitors.

NO Inhibitors	Substances
Arginine analogue	L-NMMA(Nω-monomethyl-L-arginine), L-ADMA (NωNω-dimethyl-L-arginine), L-NAME (Nω-nitro-L-arginine methyl ester), L-NNA (Nω-nitro-L-norarginine), L-NA (NG-nitro-L-arginine), Nω-amino-L-arginine, NOLA, LNIO (Nω-imminoethyl-L-ornithine), L-canavanine, D-arginine, L-glutamate
NADPH inhibitors	diphenyleneiodonium
Calmodulin antagonist	trifluoperazine, chlorpromazine, calmidazolium, W7, W13
Inhibition of BH4 synthesis	DAHP, methotrexate, N-acetyl-5-hydroxytryptamine
Feedback inhibition	NO
Substances that react with heme	carbon monoxide, methylene blue

## Data Availability

Not applicable.
